# The challenge of detecting genotype-by-methylation interaction: GAW20

**DOI:** 10.1186/s12863-018-0650-7

**Published:** 2018-09-17

**Authors:** Mariza de Andrade, E. Warwick Daw, Aldi T. Kraja, Virginia Fisher, Lan Wang, Ke Hu, Jing Li, Razvan Romanescu, Jenna Veenstra, Rui Sun, Haoyi Weng, Wenda Zhou

**Affiliations:** 1 Division of Biomedical Statistics and Informatics, Department of Health Sciences Research, Mayo Clinic, 200 First St. SW, Rochester, MN 55905 USA; 2 Division of Statistical Genomics, Department of Genetics, Center for Genome Sciences and Systems Biology, Washington University School of Medicine, 660 Euclid Ave, Saint Louis, MO 63110 USA; 3 Department of Biostatistics, Boston University School of Public Health, Boston, 715 Albany St, Boston, MA 02118 USA; 4 Department of Electrical Engineering and Computer Science, Case Western Reserve University, 10900 Euclid Ave, Cleveland, OH 44106 USA; 5 Lunenfeld-Tanenbaum Research Institute, Sinai Health System, University of Toronto, 600 University Ave, Toronto, ON M5G 1X5 Canada; 6 Department of Biology, Dordt College, 498 4th Ave. NE, Sioux Center, IA 51250 USA; 7 Department of Mathematics and Statistics, Dordt College, 498 4th Ave. NE, Sioux Center, IA 51250 USA; 8 Division of Biostatistics, Centre for Clinical Research and Biostatistics, JC School of Public Health and Primary Care, the Chinese University of Hong Kong, Shatin, N.T, Hong Kong, SAR China; 9 CUHK Shenzhen Research Institute, Shenzhen, China; 10 Department of Statistics, Columbia University, 1255 Amsterdam Avenue, New York, NY 10027 USA

**Keywords:** Methylation, Interaction, Genome wide association, Region based association, Candidate gene association, Mediation analysis, Multi-level Gaussian model, LASSO, Adaptive W-test, Regression and random forest trees

## Abstract

**Background:**

GAW20 working group 5 brought together researchers who contributed 7 papers with the aim of evaluating methods to detect genetic by epigenetic interactions. GAW20 distributed real data from the Genetics of Lipid Lowering Drugs and Diet Network (GOLDN) study, including single-nucleotide polymorphism (SNP) markers, methylation (cytosine-phosphate-guanine [CpG]) markers, and phenotype information on up to 995 individuals. In addition, a simulated data set based on the real data was provided.

**Results:**

The 7 contributed papers analyzed these data sets with a number of different statistical methods, including generalized linear mixed models, mediation analysis, machine learning, W-test, and sparsity-inducing regularized regression. These methods generally appeared to perform well. Several papers confirmed a number of causative SNPs in either the large number of simulation sets or the real data on chromosome 11. Findings were also reported for different SNPs, CpG sites, and SNP–CpG site interaction pairs.

**Conclusions:**

In the simulation (200 replications), power appeared generally good for large interaction effects, but smaller effects will require larger studies or consortium collaboration for realizing a sufficient power.

## Background

Genes do not act in isolation: the expression of gene-coded proteins is affected by many factors, including other genes, environment, and epigenetics. These interactions can complicate the identification of genes related to a particular trait or disease because some genes with an important effect through interaction can have a small marginal effect. The usefulness of interaction models for identifying causation based on disease and risk factors is demonstrated by Ruth Othman’s 1996 seminal paper [1]. As a result of recognition of the importance of interactions, a variety of methods that incorporate it were developed, especially in the case of gene–environment interaction. These methods include logistic [2] and generalized linear models [3], and machine learning [4].

This paper surveys the findings of the papers presented at GAW20 in group 5: Gene by Methylation Interactions. Even though there are issues common to all interaction approaches, each interaction type has its own specific features. In the case of gene by epigenetic interactions, all the mechanisms of epigenetics are not yet fully understood. However, it is generally accepted that methylation of a DNA strand causes loci on the methylated span to be turned “off.” The generally available measure of methylation is the percentage of copies of a DNA strand site that are methylated (generally in a single individual). The expectation is that individuals with higher levels of methylation at a site will have a lower level of gene expression for that site. Incorporating this expectation into a statistical analysis can simplify it, as compared to, for example, an analysis of an environmental factor for which the direction and mechanism of the effect are completely unknown. As our understanding of the relationships between epigenetics and genes, environment, and ancestral epigenetics improves, we will be able to further refine the statistical tests for gene by epigenetic interaction. The papers included in this group represent a wide range of approaches facilitated by the current understanding of gene by epigenetic interaction.

The data for GAW20 was provided by the Genetics of Lipid Lowering Drugs and Diet Network (GOLDN) project [5, 6]. GAW20 also provided simulated data with similar characteristics to the real data. While each group gave the data a different treatment, using the same underlying data facilitated comparison between the methods of the different groups. Most of the papers included in this group focused on methods to directly detect gene by epigenetic interaction effects, but some also explored related issues. The overall aim was to analyze the GAW20 data via different methods and compare their usefulness in the discovery of new/previously reported (with real data) or of expected findings (simulated data).

## Methods

The GAW20 group 5 applied methods to both available types of data: real and simulated [7]. The real data set consisted of phenotypes of high-density lipoprotein cholesterol, triglyceride (TG) levels, and metabolic syndrome diagnosis, before and after treatment with fenofibrate, genome-wide methylation pre- and posttreatment, and dense genome-wide single-nucleotide polymorphisms (SNPs) from the GOLDN study. The simulated data set, which was based on the real data, included 200 replicates of posttreatment TG and methylation levels. The subjects in the GOLDN study, who self-reported to be white, were recruited at the Minneapolis, MN, and Salt Lake City, UT Family Heart Study centers. The GOLDN study was designed with 2 arms: a high-fat meal challenge and fenofibrate treatment for 3 weeks to identify genetic determinants of lipid response to 2 interventions. A total of 1053 individuals met all eligibility requirements, while the data in use for lipids at visits 1 and 2, at the start of the trial, and at visits 3 and 4, at the end of the trial, that have genotype and methylation were smaller [8–12]. The GAW20 simulated data [7] followed the structure of real data, by simulating the effects of a lipid-lowering medication in interaction with methylation by affecting TG levels. A number of major variants, as well as a large number of background variants, at the genotype and methylation levels were causative of changing TG levels at visits 3 and 4. Three papers from the group studied changes on TG (Daw et al., Fisher et al., and Sun et al). Sun et al. differ from the other two in that they use percent change and dichotomized if the treatment was effective or not. Four papers look at the average TG levels either TG_pre_ or TG_post_ (Daw et al., Romanescu et al., Veenstra et al., and Zhao and Lo). One paper examined cytosine-phosphate-guanine (CpG) site variation (Hu and Li).

Most papers examined real GOLDN (CpG site, SNP, and/or TG) data. One paper analyzed the simulated data in addition to real data (Sun et al), and one paper studied simulated data only (Daw et al.; Table 1). The methods employed for detecting epigenetic by genetic interactions/main effects included generalized linear model (GLM), mixed model, regression tree model, random forest [13], mediation interaction model [14], gene-based test [15], W-test [16], candidate gene approach [16] and LASSO (least absolute shrinkage and selection operator)-type regression [17] techniques. Additionally, Gaussian mixture models were applied to examine the relationship between methylation and genotype. For a full description of each method, see the appropriate paper.

**Table 1:** Paper, data used, and methods implemented for capturing genetic, epigenetic, and associations with phenotypes

No	Paper	Data used	GAW20 simulations Answers known	Methods	Specific data and modeling	Response variable
1	Daw et al	Simulated	YES	GLM Linear mixed models Regression trees Random forest	Simulated data (200 replications) Simulated effect sites + noise	ave(log(TG_post_)) ave.(log(TG_post_)) − ave.(log(TG_pre_))
2	Fisher et al	Real data	N/A	Mediation interaction model	GWAS EWAS pairs with P_1_ < 10^−3^ and P_2_ < 0.05 The effect of change in methylation on change in TG differs by genotype Power: simulation with 500 replications	logTG_post_ − logTG_pre_
3	Hu and Li	Real data	N/A	Gaussian mixture-model clustering Gaphunter a thresholding-based method	CpG × SNP (GW) multimodal CpG sites Association of CpG identified with overlapping SNP Association with nearby SNPs	Pretreatment CpG Posttreatment CpG
4	Romanescu et al	Real data	N/A	Regional-based test	Single CpG association (CH11) Regional SNP PC association (CH11) Regional CpG PC association (CH11) Regional SNP + regional CpG association (CH11)	ave(ln(TG_pre_))
5	Sun et al	Both	YES	Adaptive W-test with 2000 repetitions to get Type I error	In both analysis main and interaction associations Simulation: candidate pairs and noise pairs Real data: (CH11)	delta(TG%) = 1, if delta(TG%) > 30% = 1, otherwise 0 delta(TG%) = (TG_pre_ − TG_post_)/TG_pre_
6	Veenstra et al	Real data	N/A	Lmekin of coxme, lm in R	(1) SNP association + (2) CpG association (1) + (2) + CpG × SNP	Ave(TG1 + TG2) baseline HDL
7	Zhou and Lo	Real data	N/A	Group LASSO-regression, kinship matrix, stability selection	CpG association (GW) SNP association (GW) (1) + (2) + CpG × SNP (CH11)	Ave(TG)

Abbreviations: *CH11* chromosome 11, *CpG* cytosine-phosphate-guanine, *EWAS* epigenome-wide association study, *GLM* generalized linear model, *GW* genome-wide, *GWAS* genome-wide association study, *HDL* high-density lipoprotein cholesterol, *PC* principal components; pre, pretreatment; post, posttreatment, *SNP* single-nucleotide polymorphism, *TG* triglyceride

## Results

Several methods were used to detect SNP by methylation interactions using either simulated or real data. Some of the real data papers conducted a genome-wide association study, whereas others focused on either candidate genes or chromosome 11 where a previous finding in the provided data was reported (see Table 1). Consequently, for the real data papers we focused on comparing results on chromosome 11 **(**Table 2). For assessing results in the simulated data, we can simply compare to the known answers.

**Table 2:** Chromosome 11 findings from real data analyses

No	Approximate position (MBs)	Gene(s)	Papers	Legend No	Paper
1	0.6	*C11orf63*	5	1	Daw et al
2	1.9	*TNNT3*	5	2	Fisher et al
3	5.6	*TRIM5,TRIM6-TRIM34,TRIM3*	5	3	Hu and Li
4	15.7	*rs900865*	5	4	Romanescu et al
5	19.4	*NAV2*	5	5	Sun et al
6	26.5	*rs4347345*	5	6	Veenstra et al
7	30.4	*MPPED2*	5	7	Zhou and Lo
8	40.1	*LLRC4C*	4		
9	57.3	*SLC43A1*	4		
10	61.6	*FADS1, FADS2, FADS3*	6		
11	68.6	*CPT1A* ^*a*^	4,7		
12	77.1	*CUCY2E*	5		
13	102.8	*LOC100128088*	5		
14	114.1	*ZBTB16*	5		
15	116.7	*APOA5, APOA1, BUD13, AP006216.5* ^*a*^	4,6,7		
16	133.4	*B3GAT1*	5		

^a^Genes on chromosome 11 that were identified in more than one paper from the GAW group5

In Daw et al. [13], GLMs, mixed models, regression trees, and random forest models were applied to 200 replications of simulated data. PROC GLM and PROC MIXED of SAS were used for the linear models, taking into account the family structure as a random effect. Four models were used: model 1.a included the intercept, the main effects, CpG sites and SNPs, and their interaction effect on TG post–medication-treatment plus a vector of covariates (age, age^2^, sex, and the average of log[TG_1_] and log[TG_2_] before treatment); model 1.b included only the interaction effect on TG post–medication-treatment plus the vector of covariates; model 2.a included as the response variable the change of TG (posttreatment–pretreatment) including the main effects, SNP and the methylation difference between visits 4 and 2, their interaction and covariates (age, age^2^, sex); model 2.b was a reduced version of model 2.a that included only interactions. The models 1.a and 1.b were fitted using PROC GLM and PROC MIXED. Models 2.a and 2.b were fitted using only PROC MIXED. Regression trees were applied using SAS (v. 9.4) and SAS Data Mining (v. 14.1), as well as random forest in Python from the SciKit-Learn package, to predict outcome variables by averaging the predictions of a large number of uncorrelated regression trees. The authors tested for sensitivity using causative SNP-CpG site pairs and noncausative SNP-CpG site pairs to estimate the empirical null. They identified reasonable power to detect the main causative loci, depending on sample size and strength of the effects at the SNP and CpG sites.

Sun et al. [16] worked with both simulated data and real data. They analyzed only the 84th replication of GAW20 simulated data. This replicate was recommended as the one to use for comparing results with other teams when not working with all 200 replicates. Their W-test [18] checks for pairwise epistasis based on the null hypothesis that the joint distribution of a set of SNPs has no difference in the cases versus controls. They tested the gene-methylation epistasis in subjects’ posttreatment using an additive model. Individuals’ treatment response was dichotomized based on change from pre-TG to post-TG levels. A change greater than 30% [19] was defined as effective; a lesser change was defined as ineffective. For the W-test, methylation was dichotomized into high and low groups. Thus, the SNP-CpG site pair forms a genetic combination of 6 categories. In addition, Sun et al. examined logistic regression models with both dichotomized and continuous CpG sites [16]. In their application of 3 tests to 1 simulated replicate, they examined 10 sites: 5 “causative” and 5 “noise.” None of the “noise” sites were significant for any of the tests. Three of the 5 “causative” sites were significant for all 3 tests, and 1 “causative” site was marginally significant for W-test and for the 2 logistic regression tests. The fifth site was not detected using any of the 3 methods. In real data they focused on chromosome 11, and applied only their W-test. Their top 15 hits had *p* values from 7.5 × 10^− 6^ to 7.0 × 10^− 5^. None of these 15 were reported by other papers in our group.

Mediation analysis was implemented by Fisher et al. [14], who investigated change in pretreatment to posttreatment TG levels as outcome by considering the hypothesis that genotype affects change in TG level by means of its effects on methylation. Thus, the effect of change in methylation on change in TG differs by genotype. For their analysis, they used a real data sample of 406 individuals with complete genotype, methylation, TG levels, and covariate data, performing estimation with the *coxme* R package [14]. They conducted a genome study, but none of their reported hits were on chromosome 11. They also had no hits in common with Veenstra et al., who also conducted genome-wide analysis. Fisher et al’s power calculations suggest that the available data were underpowered for their method.

Hu and Li investigated the relation between genotype and methylation levels pretreatment and posttreatment via Gaussian mixture-model clustering. First, they identified CpG sites with multimodal methylation level distributions (mmCpGs). Subsequently, they identified SNPs that segregate with mmCpG modes to identify genes that may play a role in methylation levels. The authors identified 3785 and 3847 mmCpGs in pretreatment and posttreatment data sets [20]. Of these, 453 directly overlap with SNPs. Some show strong correlation between genotype and methylation levels, which suggests a relation between epigenetics and genetics that may be modeled in the statistical analysis.

Romanescu et al. applied a region-based test to identify CpG site regions associated with TG levels. They applied 4 statistical models: a single-site test for CpG site association and 3 regional tests. In regions defined by GENCODE annotation, they conducted a principal component analysis for SNP genotypes and for methylation levels. Then they conducted association tests incorporating (a) the SNP-principal component, (b) the CpG sites principal component, and (c) both. The authors used chromosome 11 real data. Their mixed linear models account for family structure, while incorporating the method proposed by Gauderman [21] using principal components on SNPs and CpG site regions [15]. They identified 4 regions on chromosome 11, including 2 that were identified in other studies: the *CPT1A* and the *APOA5* regions.

Veenstra et al. employed a candidate gene and a genome-wide approach to identify genetic and epigenetic effects on baseline TG. To remove any confounding effects, they adjusted for 6 covariates: age, sex, smoking, metabolic syndrome diagnosis (msdx), fasting time at baseline, and high-density lipoprotein cholesterol. They used the *lmekin* function from the *coxme* R package [22]. Furthermore, they analyzed the residual TG, after adjusting for these covariates, by modeling main effects of SNPs, CpG sites, and their interactions for approximately 700,000 pairs using an F-test. Additionally, they carried out the same procedure on 18 gene regions containing 423 unique SNP-CpG sites. Their genome-wide analysis identified no results on chromosome 11. In contrast, their candidate gene analysis, which included 7 genes in 2 regions on chromosome 11, found them as significant in both regions. One of the regions, *APOA5*, was also found in other papers of this group.

Zhou and Lo applied LASSO (least absolute shrinkage and selection operator)-type regression models to identify SNP–CpG site interactions [17]. The methods included the group LASSO approach for categorical variables and interactions [23]. These coauthors analyzed whole-genome CpG site association and whole-genome SNP association, as well as interaction modeling with main effects on chromosome 11 only. Zhou and Lo implemented a stability selection method, which quantified the uncertainty in variable selection, thus controlling the family-wise error rate. They identified 2 regions on chromosome 11 that were also identified by other papers in this group, including *CPT1A*.

## Discussion

The contributing papers in the Gene by Methylation Interaction group employed 10 major methods to capture such interaction. Their results suggest that a variety of methods can be employed to productively analyze combined genetic and epigenetic data. The choice of response variable was mostly pre(log(TG)); 2 papers looked at absolute change in log(TG), with 1 paper also examining post(log(TG)) and 1 dichotomizing percent change in TG. In addition, 1 study used CpG sites as a response variable. Papers 4, 6, and 7 all analyzed baseline TG and obtained a set of overlapping results on chromosome 11 (see Table 1). One of these results was *CPT1A*, which was previously reported by the GOLDN study [6]. This overlap of results lends credence to the accuracy of the results. Furthermore, the 2 regions of overlap, *CPT1A* and the genes in the *APOA5* region, are biologically involved in lipid metabolism. The other papers that analyzed real TG data (2 and 5; see Table 1), analyzed measures in TG between pretreatment and posttreatment. This difference in phenotype may explain why the 2 changes in real TG papers do not replicate the findings of the TG-baseline papers. Furthermore, these 2 last papers use different measures of change in TG, which might explain the lack of replication between the two. Although we have summarized the reported results in the real data papers, each paper used slightly different criteria for reporting results.

For evaluating power, the simulated data, in which the answers are known, are more suited than the real data. The GAW20 GOLDN real data and GAW20 simulated data were similar in sample size, less than 900 individuals in baseline and less than 700 individuals in visits 3 and 4, thus making for samples that limit the power of discovery of causative variants. The Daw et al. paper shows that in the GAW20 simulated data there was a reasonable power to detect causative loci, but detection of background causative rare alleles with very small contributions is difficult with this sample size. Fisher et al. report the need for a sample size of approximately 19.5 K to be able to detect significant indirect effects. Thus, it is reasonable to expect that methods to detect gene by methylation interaction will yield better results in many large studies.

The analyses used employed various types of linear models. The Daw et al., Fisher et al., Sun et al., Veenstra et al., and Zhou and Lo papers all include models with SNP by methylation interaction, while the paper by Romanescu et al. includes a gene-region model that comprises effects for the principle components of both SNP genotype and methylation within a single gene-region. These models appear reasonable, given the current understanding of genetics and epigenetics. However, the paper of Hu and Li found some correlations between measured CpG site levels and SNP genotypes, which may indicate additional relationships that should be incorporated as our understanding improves. The methods applied in these papers have clear utility, but improvements may be possible as new relationships between genetic and epigenetic data are discovered and incorporated into the models.

## Conclusions

The SNP–methylation interaction group provides very creative, interesting approaches for application to the GAW20 data sets, using either the GOLDN or simulated data. Several papers found a number of causative SNPs using the simulation set and the previous results on chromosome 11 were confirmed. Other papers identified different SNPs, CpG sites, and SNP–CpG interaction sites. Power appeared generally good for large interaction effects for these methods in a sample similar in size to the one provided (approximately 500 to 1000 individuals), but smaller effects appear to require larger studies or consortiums to have sufficient power.

## Abbreviations

CpG: Cytosine-phosphate-guanine
GAW: Genetic Analysis Workshop
GLM: Generalized linear model
GOLDN: Genetics of Lipid Lowering Drugs and Diet Network
LASSO: Least absolute shrinkage and selection operator
mmCpGs: CpGs with multimodal methylation level distributions
msdx: Metabolic syndrome diagnosis
SNP: Single-nucleotide polymorphism
TG: Triglyceride
